# A Brazilian regional basic diet-induced chronic malnutrition drives liver inflammation with higher ApoA-I activity in C57BL6J mice

**DOI:** 10.1590/1414-431X20209031

**Published:** 2020-05-08

**Authors:** M.J.S. Santos, K.M. Canuto, C.C. de Aquino, C.S. Martins, G.A.C. Brito, T.M.R.P. Pessoa, L.R. Bertolini, I. de Sá Carneiro, D.V. Pinto, J.C.R. Nascimento, B.B. da Silva, J.T. Valença, M.I.F. Guedes, J.S. Owen, R.B. Oriá

**Affiliations:** 1 Laboratório da Biologia da Cicatrização Tecidual, Ontogenia e Nutrição de Tecidos, Departamento de Morfologia e Instituto de Biomedicina, Faculdade de Medicina, Universidade Federal do Ceará, Fortaleza, CE, Brasil; 2 Núcleo de Microscopia e Processagem de Imagens, Departamento de Morfologia e Instituto de Biomedicina, Faculdade de Medicina, Universidade Federal do Ceará, Fortaleza, CE, Brasil; 3 Laboratório de Biologia e Biotecnologia Molecular, Universidade Estadual do Ceará, Fortaleza, CE, Brasil; 4 Division of Medicine, Royal Free Campus, University College London Medical School, Hampstead, London, United Kingdom; 5 Ciências da Saúde, Universidade de Fortaleza, Fortaleza, CE, Brasil; 6 Departamento de Patologia, Faculdade de Medicina, Universidade Federal do Ceará, Fortaleza, CE, Brasil

**Keywords:** Liver, Hyperlipidemia, Malnutrition, Inflammation, Kupffer cells, Apolipoprotein A-I

## Abstract

Malnutrition is still considered endemic in many developing countries. Malnutrition-enteric infections may cause lasting deleterious effects on lipid metabolism, especially in children living in poor settings. The regional basic diet (RBD), produced to mimic the Brazilian northeastern dietary characteristics (rich in carbohydrate and low in protein) has been used in experimental malnutrition models, but few studies have explored the effect of chronic RBD on liver function, a central organ involved in cholesterol metabolism. This study aimed to investigate whether RBD leads to liver inflammatory changes and altered reverse cholesterol metabolism in C57BL6/J mice compared to the control group, receiving a standard chow diet. To evaluate liver inflammation, ionized calcium-binding adapter protein-1 (IBA-1) positive cell counting, interleukin (IL)-1β immunohistochemistry, and tumor necrosis factor (TNF)-α and IL-10 transcription levels were analyzed. In addition, we assessed reverse cholesterol transport by measuring liver apolipoprotein (Apo)E, ApoA-I, and lecithin-cholesterol acyltransferase (LCAT) by RT-PCR. Furthermore, serum alanine aminotransferase (ALT) was measured to assess liver function. RBD markedly impaired body weight gain compared with the control group (P<0.05). Higher hepatic TNF-α (P<0.0001) and IL-10 (P=0.001) mRNA levels were found in RBD-challenged mice, although without detectable non-alcoholic fatty liver disease. Marked IBA-1 immunolabeling and increased number of positive-IBA-1 cells were found in the undernourished group. No statistical difference in serum ALT was found. There was also a significant increase in ApoA mRNA expression in the undernourished group, but not ApoE and LCAT, compared with the control. Altogether our findings suggested that chronic RBD-induced malnutrition leads to liver inflammation with increased ApoA-I activity.

## Introduction

Malnutrition is still endemic in many parts of the developing world where poverty coexists with infectious diseases, much of it associated with precarious sanitation and hygiene (1). Malnutrition is a health issue particularly in growing children, especially in the first two years of life, when they undergo rapid cognitive and physical development (2,3). Very often malnutrition increases the risk for enteric infections and vice-versa (4), so that the condition may create a self-amplifying vicious cycle that impairs growth and brain function by reducing intestinal nutrient absorption and increasing nutrient loss (5). Prolonged infection/malnutrition per se may increase daily nutrient requirements much more than the needs of a healthy child. A metabolomics study from Preidis and colleagues has shown that neonatal malnutrition leads to liver inflammation and increased oxidative stress in suckling mice (6). In addition, Wistar rats receiving a low protein diet show growth impairment, edema, liver steatosis, hypoalbuminemia, and anemia, which are common characteristics of human kwashiorkor (7). In many developing countries, the composition of diets available to weanling children feature both low protein and high carbohydrate concentrations (8,9), including the Brazilian semi-arid northeast, one of the poorest regions in the country (10). Our group has utilized the regional basic diet (RBD), enriched in carbohydrate and with low protein concentration, to induce experimental malnutrition with disrupted intestinal barrier function (11) and then assessed intestinal inflammatory markers (12). Chronic malnutrition-driven stunting has been recognized to be associated with low-grade systemic inflammation in children (13).

A high-carbohydrate diet may overload liver functioning and amplify the low-grade inflammatory insult due to chronic low-protein malnutrition. Chronic malnutrition/infection states early in life may predispose to obesity and metabolic syndrome later (14). The mechanisms are mostly unknown but may involve long-term changes in liver metabolic function. Indeed, the effect of malnutrition on hepatic reverse cholesterol transport has not been explored. In this study, we have addressed whether the RBD, as a model of chronic malnutrition in weanling mice, could have a pro-inflammatory effect on the liver, with a focus on reverse cholesterol transport, which may have long-term consequences on liver metabolism with aging.

## Material and Methods

### Animals

C57BL6J mice weighing 5–8 g were obtained from the State University of Ceará vivarium and housed in polypropylene boxes with free access to diet and water, under controlled temperature and humidity, and 12-h light/dark cycle in ventilated boxes. Immediately after weaning (21 days of life), experimental mice were randomly divided into two groups. A nourished group received a standard chow diet and the other group received an isocaloric RBD for forty days. The body weights were monitored every three days. RBD is a well-studied rodent diet high in carbohydrates and marginally deficient in protein, fat, and minerals. It was formulated according to Teodósio et al. (10) to represent the multideficient diet of poor populations in northeastern Brazil. RBD and commercial chow (Puro lab 22PB^®^, Brazil) diets present the following nutrients, respectively: protein (7 and 22%), carbohydrate (88 and 65%), and fat (5 and 15%) (Table 1). All experimental mice were euthanized by decapitation on post-natal day 61, after being previously anesthetized with ketamine (8 mg/100 g) and xylazine (0.8 mg/100 g).

**Table 1:** Nutritional composition (g/g%) of the multideficient regional basic diet (RBD) and the standard commercial control diet.

	Standard commercial diet	Regional basic diet
Proteins	22.00	7.00
Carbohydrates	65.00	88.00
Lipids	15.00	5.00
Fibers	7.00	6.25
Minerals		
Ca^2+^	1.20	0.65
Mg^2+^	0.32	0.44
K^+^	0.80	0.54
Na^+^	0.27	0.25
Humidity	12.00	13.00
kcal/100g	407.10	408.10

All experimental protocols were in compliance with the Brazilian College for Animal Experimentation (COBEA) and the Institutional Animal Care and Use Committee guidelines from the State University of Ceará.

### Histopathological scores

Slides containing liver hematoxylin and eosin-stained 5-µm-thick sections were used for histopathological analyses. To evaluate non-alcoholic hepatitis, a histopathological scoring was used as described by Kleiner et al. (15) and the Pathology Subcommittee of the NASH Clinical Research Network. The samples were analyzed by a trained pathologist, blinded to the research groups, according to Table 2. Cell ballooning was measured by the following scores: 0) no liver cells showing ballooning; 1) few cells; and 2) various liver cells showing prominent ballooning.

**Table 2:** Histopathological scoring according to Kleiner et al. 15, depending on the extension and involvement of the hepatic parenchyma.

Degree	Parenchymal impairment
0	Absent (up to 5%)
1	Low (5 to 33%)
2	Moderate (34 to 66%)
3	High (More than 66%)

### Serum alanine aminotransferase

Blood obtained by animal decapitation was transferred to Eppendorf tubes and allowed to coagulate at room temperature. The serum tubes were centrifuged (xxxx *g*) at 4°C for 3 min to separate the serum from the clotting debris. The sera were then stored in a freezer at −20°C for further serum alanine aminotransferase (ALT) analyses. ALT is a well-known biomarker of liver function. We used the Reitman and Frankel method for analysis (kit for colorimetric assay - LABTEST^®^, Brazil).

### qRT-PCR analyses

The liver tissue (30 mg) was macerated in Trizol reagent (Invitrogen^®^, USA) with the aid of the Tissueruptor (Qiagen^®^, USA). For complete RNA extraction, the manufacturer's instructions were followed (RNAse mini extraction kit, Invitrogen^®^).

To prepare the cDNA, 1 μg of total RNA, treated with DNAse (Ambion^®^, USA), was used. Primers oligo (dT), dNTPs, and Super Script III enzyme were used following the manufacturer's instructions (Invitrogen^®^). RNA quantification was done using Nanodrop equipment (Thermo^®^, USA). The qPCR reactions were performed with three biological replicates for each treatment. qRT-PCR was performed on StepOne Plus (Applied Biosystems^®^, USA) equipment, with the following schedule: activation of the Taq DNA polymerase enzyme for 5 min at 94°C, 40 cycles of denaturation at 94°C for 30 s, annealing at 60°C, and extension at 72°C for 30 s, with the collection of fluorescence data at each cycle. The amplification efficiency of the PCR was calculated from the slope of the linear regression equation obtained by the analysis software, according to the following equation: E = 10 (−1 / slope). The gene expression data, representative of the three independent biological replicates for each treatment, were submitted to analysis of variance. The means were compared and considered significantly different at 5% probability. All reactions were performed in duplicate and normalized by threshold fluorophore. For interleukin (IL)-10 and tumor necrosis factor (TNF)-α, CT values were normalized by the level of β-actin. For the relative quantification of ApoA1, ApoE, and LCAT, RPLP0 was used as the reference gene.

Ten microliters of Master Mix (SYBR Green Real-Time PCR Master Mix, Life Technologies^®^, USA), 5 μL of cDNA, 2 μL of oligonucleotides, and 3 μL of ultrapure water were used in the reactions. The oligonucleotides used were synthesized (Life Technologies^®^) from sequences deposited in a specific database (16). The sequences of the oligonucleotides used are found in Table 3.

**Table 3:** Primers used for the RT-qPCR analyses.

Gene	Forward	Reverse	Genebank reference
APOE	CTTCTGGGATTACCTGCGCTGG	GTAGATCCTCCATGTCGGCT	NM_009696
APOA-1	TCAAAGACAGCGGCAGAGAC	CACCTTCTGGCGGTAGAGCTC	NM_009692
LCAT	CTGGCTCCTCAATGTGCTCTTC	AGGCCGTGTGTGGTTACTGAGT	NM_008490
RPLP0	GCTTCATTGTGGGAGCAGACA	CATGGTGTTCTTGCCCATCAG	RTPrimerDB ID 1261
IL-10	AAAGCAAGGCAGTGGAGCAG	TCAAACTCATTCATGGCCTTGT	NM_012854
TNF-α	TCGAGTGACAAGCCCGTAGC	CTCAGCCACTCCAGCTGCTC	HQ 201305.1
β-actin	CCCTGGCTCCTAGCACCAT	sGAGCCACCAATCCACACAGA	NM_031144.3

### Immunohistochemistry

Immunohistochemistry for IBA-1, ApoA-1, and IL-1β was performed using the streptavidin-biotin-peroxidase method. The tissues were dehydrated in alcohol and then included in paraffin. Serial sections of 4 μm were placed on L-polylysine-coated slides, suitable for immunohistochemistry. The sections were dewaxed, hydrated in xylene and alcohol, and immersed in 0.1 M citrate buffer (pH 6.0) in a water bath for 20 min for antigen recovery at 65°C. After cooling at room temperature for 20 min, sections were rinsed with phosphate-buffered saline (PBS), and blocked with endogenous peroxidase with 3% H_2_O_2_ solution (20 min). Protein blocking was then performed with 5% BSA (bovine serum albumin) for 40 min. The sections were incubated overnight with goat anti-IBA1 primary antibody (ABCAM^®^, USA) diluted 1:100 in antibody diluent. After PBS washing, sections were incubated with biotinylated goat IgG diluted 1:400 (Santa Cruz^®^, USA) for 30 min. After washing, the sections were incubated with streptavidin conjugated peroxidase complex (Santa Cruz^®^ ABC complex) for 30 min. After further washing with PBS, sections were stained with chromogen 3,3'diaminobenzidine peroxide (DAB) and counterstained with Mayer's hematoxylin and cover-slipped. Negative controls were processed simultaneously as described above, with the primary antibody being replaced with 5% PBS-BSA.

For IBA-1 positive cell counting, 10 magnification fields (×400) per histological section (each mouse) were used. Immunohistochemistry images were captured using a light microscope (Leica, Germany) coupled to a camera with LAZ 3,5 acquisition system (Leica DM1000). Positive cells were counted from high resolution-digitalized images with the aid of Image J software (NIH, USA).

### Statistical analyses

Data are reported as means±SE. Two-way ANOVA was used to analyze weight gain data and the unpaired Student's *t*-test was used for the remaining analyses. P<0.05 was set to indicate significant differences.

## Results

### Body weight gain

**Figure 1:**
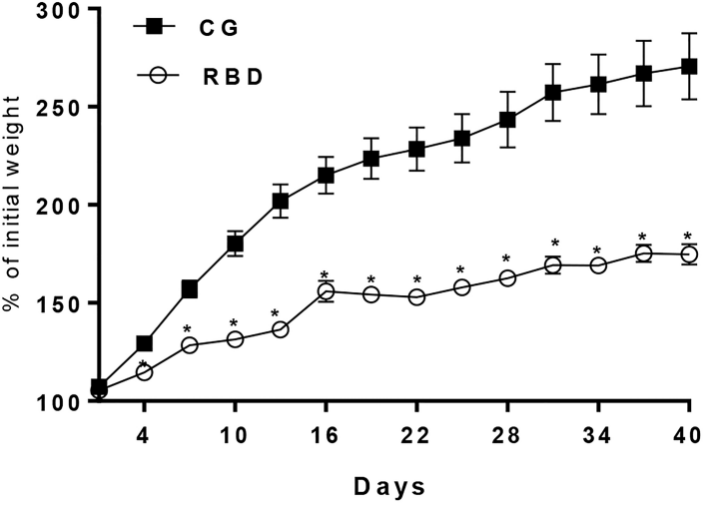
Weight gain (% of initial weight) every 3 days of C57BL6J nourished (control group, CG) and regional basic diet (RBD) undernourished mice during 40 days of dietary intervention. Data are reported as means±SE. *P<0.05, two-way ANOVA.

RBD induced a profound reduction in body weight gain compared to the nourished control group (P<0.05) as soon as the fourth day under RBD-feeding and along the remaining experimental protocol. There was no significant weight loss in the undernourished mice, but a slow weight gain pace, almost reaching a plateau. Thus, RBD feeding induced a chronic malnutrition condition (Figure 1).

### Histopathological scoring

In the histopathological analysis, no difference was found in steatosis markers (hepatocyte ballooning and micro- or macrovesicular changes) between the experimental groups, however increased hepatocyte microvesicular changes were seen more prominently in some undernourished mice (Table 4). No significant difference was found in serum ALT between groups.

**Table 4:** Histopathological liver scores in C57BL6J nourished and undernourished mice following 40 days of dietary intervention.

Groups	Scores		
	Steatosis		Ballooning
	Macrovesicular	Microvesicular	
Nourished (n=3)	0 (0)	0 (0−1)	1 (1)
Undernourished (n=6)	0 (0−1)	2 (0−3)	1 (1−2)

Data are reported as median (range). There were no significant differences between the groups (Mann-Whitney test), however, changes were more pronounced in the undernourished mice.

### Inflammatory markers

**Figure 2:**
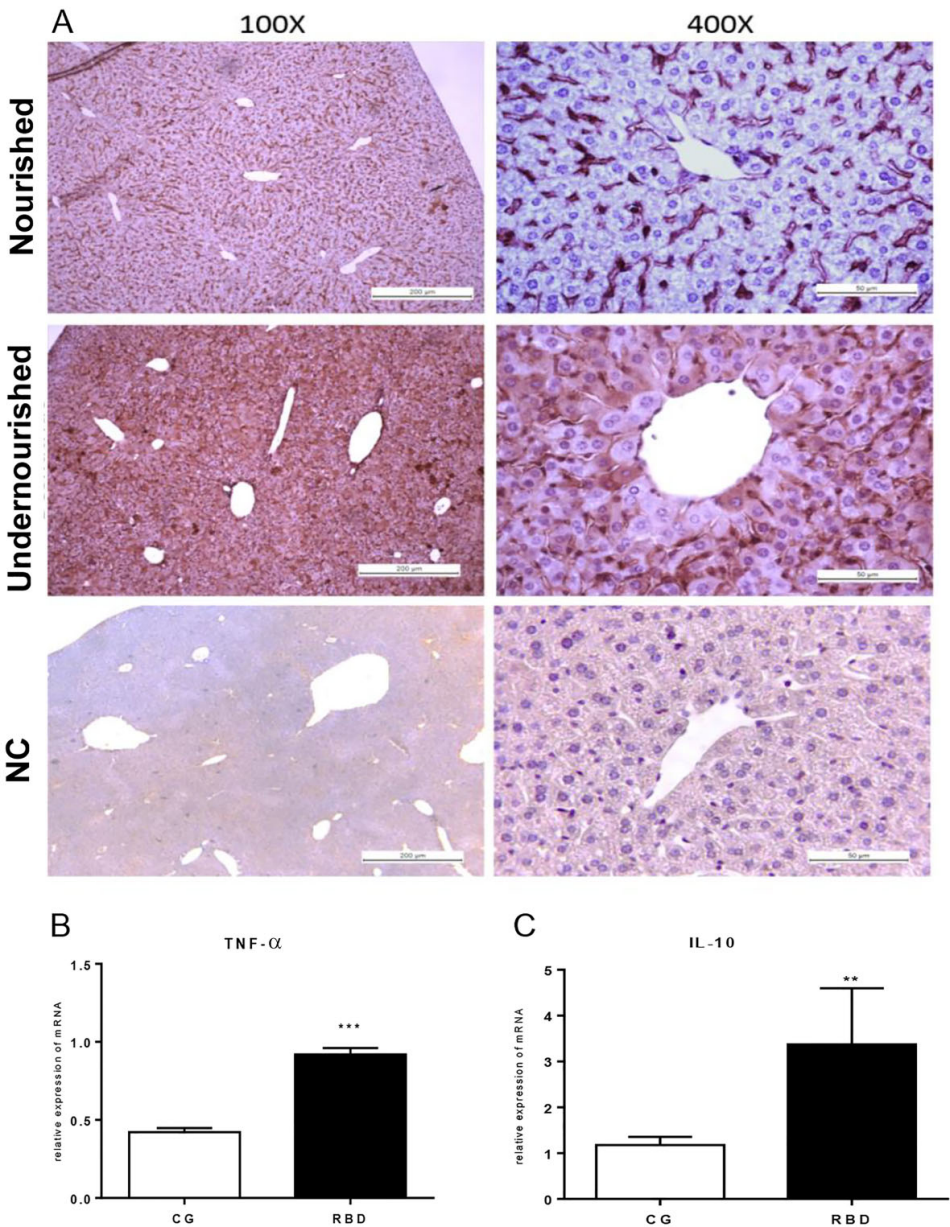
**A**, Representative panel of interleukin (IL)-1β immunostaining of liver tissue of the experimental groups after 40 days of dietary intervention. Control (nourished) and regional basic diet (RBD) groups in low (×100, scale bar 200 μm) and high magnification (×400, scale bar 50 μm). The negative control (NC) without the target antibody is also depicted. **B** and **C**, Tumor necrosis factor (TNF)-α and IL-10 transcription by RT-qPCR using β-actin as the reference gene. Data are reported as means±SE for n=6 per group. **P<0.01 and ***P<0.001, unpaired Student's *t*-test.

Significantly increased IL-1β immunostaining was observed in the liver of mice receiving RBD compared to the nourished controls. There was greater IL-1β immunostaining in the overall hepatic parenchyma, including the majority of hepatocytes (Figure 2A). In addition, prolonged feeding with RBD caused a marked increase in TNF-α (P<0.001) and reduction in IL-10 (P<0.01) transcriptional levels, compared to the controls that received the standard diet (Figure 2B and C).

**Figure 3:**
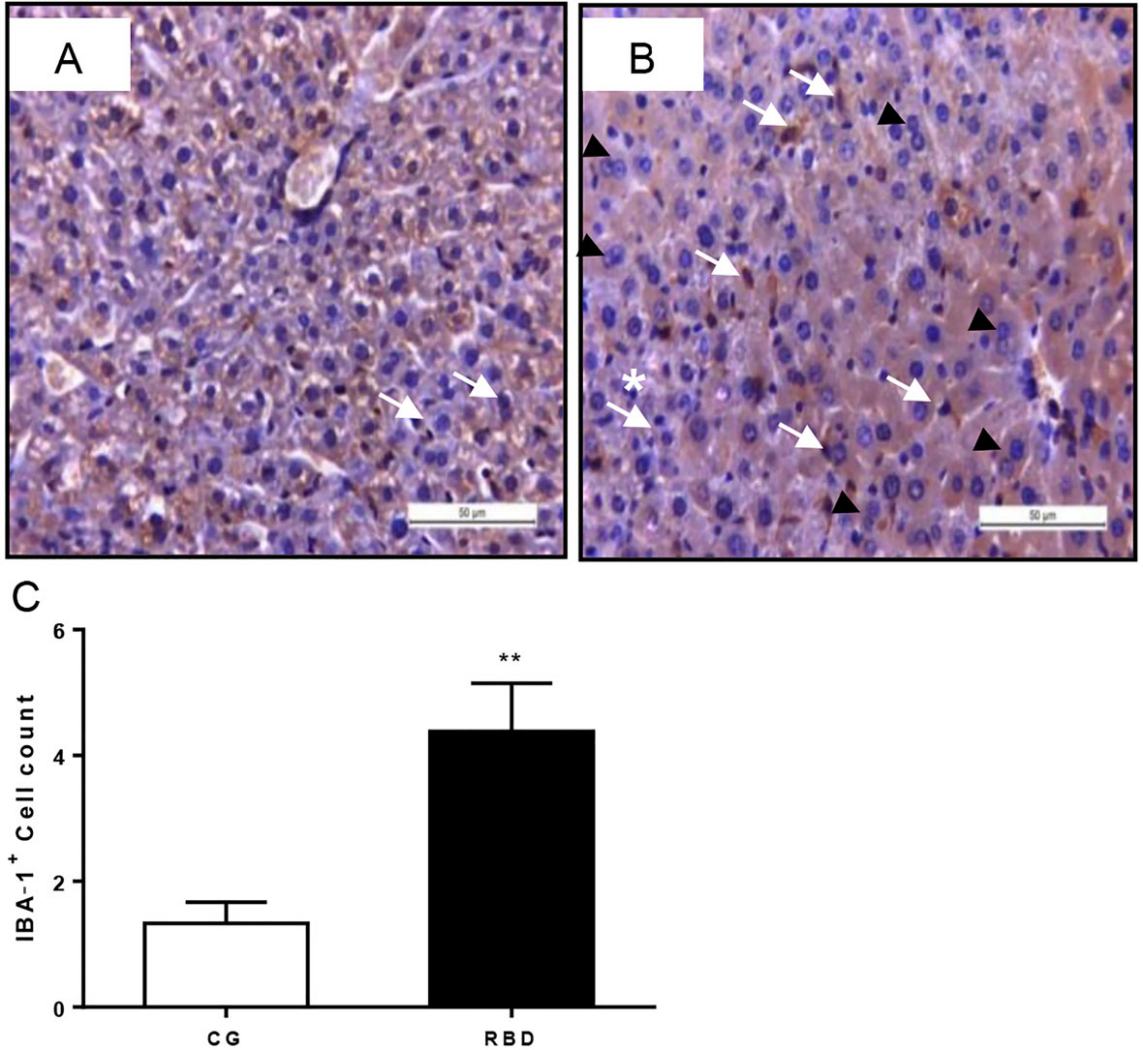
Representative liver ionized calcium-binding adapter protein-1 (IBA-1) immunostaining of the experimental groups, following 40 days of dietary intervention. **A**, Control nourished and **B**, regional basic diet (RBD) groups in high magnification (×400, scale bar 50 μm). White arrows indicate binucleated hepatocytes. Black arrowheads indicate IBA-1-positive cells. The asterisk indicates parenchyma disruption. Note lack of glycogen stores (seen by poorly stained cytoplasm in the nourished hepatic cells) and hepatocyte hypertrophy in the RBD-challenged liver. **C**, IBA-1-positive cell count. Data are reported as mean±SE for n=6 per group. **P<0.01, unpaired Student's *t*-test.

The liver from undernourished mice showed increased IBA-1 immunolabeling (depicted by increased brown staining), indicating more activated Kupffer cells (liver macrophages) (Figure 3). Furthermore, a higher count of IBA-1-positive cells was found in the undernourished group (P<0.001) (Figure 3C). Interestingly, many hepatocytes from RBD-challenged mice showed hypertrophic and polypoid (indicated by more binucleated cells) characteristics (Figure 3B).

### Reverse cholesterol transport

**Figure 4:**
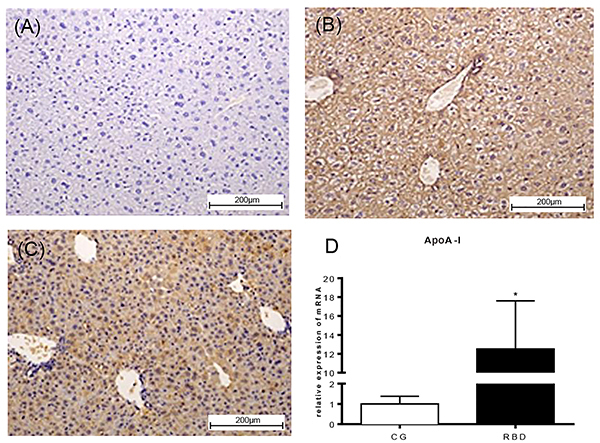
Representative liver apoA-I immunostaining of the experimental groups, following 40 days of dietary intervention. **A**, Negative control. **B**, Nourished (CG) and **C**, regional basic diet (RBD) groups (×100, scale bar 200 μm). **D**, Liver apoA-I transcriptional level from nourished and RBD-undernourished mice following 40 days of dietary intervention. Data are reported as means±SE for n=6 per group. *P<0.05, unpaired Student's *t*-test.

We assessed some markers of liver reverse cholesterol transport by analyzing ApoE, ApoA-I, and LCAT transcriptional levels. No significant change was found for ApoE and LCAT; however, there were increased ApoA-I mRNA levels in the RBD-challenged liver compared to the nourished control (P<0.05) (Figure 4D). In addition, higher ApoA-I immunolabeling was found in liver histological sections of the RBD group compared to the nourished control.

## Discussion

Several methods to induce long-term post-natal malnutrition and growth impairment have been described in the literature. Among the most commonly used are: food restriction, gradual withdrawal of the offspring during lactation (17,18), adjustment of the litter size (19), and induction of low-protein malnutrition through the use of hypoproteic diets (20). Recently, much attention has been paid to the environmental enteric dysfunction condition (EED), which may cause low-grade systemic inflammation in chronically undernourished children (21). The burdensome EED is highly prevalent in the developing world and may additionally increase the risk for metabolic disease and obesity with aging (14,22). This condition still lacks good animal models.

The RBD was developed after a food intake survey conducted by the Federal University of Pernambuco in Northeastern Brazil to evaluate the effect of intrauterine malnutrition (10). A remarkable impairment in body weight gain was observed in the RBD-challenged Wistar rats for 14 days, and the sustained weight decrease was not reversed even after zinc supplementation. In addition, RBD induced an intestinal-to-blood bacterial translocation, shown by an increased number of bacteria colony forming units in the spleen and mucosal inflammation (12).

Although RBD has been used in several studies, none of those has investigated the possible changes that this diet can cause in the liver. This is important as RBD-induced inflammatory changes in the liver could alter the reverse cholesterol transport, leading to hypertriglyceridemia and the risk for developing cardiovascular diseases. The work of Preidis and colleagues has identified important metabolic alterations in undernourished mice, subjected to neonatal maternal separation, including evidence of muscle and adipose tissue catabolism, as well as liver and bile abnormalities, oxidative stress, and inflammation (6). Findings that support that malnutrition can lead to important changes in liver cholesterol metabolism are important to guide health policies in endemic areas.

Although studies have shown elevation of ALT serum levels in children afflicted with undernutrition (7), a serum ALT increase is not a universal finding in protein-energy undernourished children (23). In the studied animal model, ALT serum levels were not significantly affected by RBD-induced malnutrition.

Increased IBA-1-positive cells and higher transcription of pro-inflammatory cytokines in the liver from RBD-challenged mice suggested increased intestinal bacterial translocation and activation of Kupffer cells by bacterial products carried out from the liver by the portal circulation. Interestingly, intestinal gram-negative bacterial overgrowth in normal rats can increase Kupffer cells reactivity to lipopolysaccharide (LPS) and reduce hepatocyte protein synthesis. When cultured alone, Kupffer cells from these animals also produced more IL-1 and prostaglandin E2 in response to LPS (24).

The increase in liver IL-10 mRNA levels may be a counterbalance response to over-inflammation, as IL-10 can reduce T-cell secretion of IL-2, lower human leukocyte antigen II expression (HLA class II), and IL-1β and TNF-α secretion by monocytes and macrophages during inflammation (25). Elevated serum IL-10 levels have been observed in newly-weaned mice receiving a 0.6% hypoprotein diet compared to controls (26).

The marked immunolabeling of IL-1β in the liver parenchyma seen in the undernourished mice corroborated with findings of increased number of IBA-1 positive cells. Interestingly, in the work by de Queiroz and colleagues, RBD induced increased jejunum levels of TNF-α, IL-1β, and IL-10 in rats, compared to the nourished group, suggesting a systemic inflammatory effect. In that sudy, zinc supplementation was able to reduce levels of TNF-α and IL-10, but not IL-1β in RBD-challenged mice compared with zinc untreated counterparts (12).

In a study with 7- and 8-week-old Swiss male rats fed either with a high-carbohydrate diet (64% carbohydrates, 19% protein, and 11% fat) or a high fat diet (45% carbohydrate, 17% protein, and 38% fat), the high-carbohydrate diet was able to raise the TNF-α plasma and liver levels (27). Likewise, in our study, chronic feeding with RBD, a diet enriched with high carbohydrate content (70.6%), compared to the standard diet (56%), increased TNF-α mRNA levels in the liver of mice.

In the liver, TNF-α is produced mainly by Kupffer cells, which are resident macrophages (28). Increased transcription of TNF-α in RBD-challenged mice is probably correlated with the increased number of Kupffer cells, as more IBA-1-positive liver cells were found, suggesting an activated inflammatory state in prolonged malnutrition.

Increased TNF-α serum levels have been documented in children with protein-energy malnutrition (29) and in the whole blood cultures from children with primary malnutrition (30), compared to well-nourished ones. In addition, Dewan et al., investigating the immune profile from 80 moderately to severely malnourished children receiving the WHO preconized diet for severe malnutrition (age one to five years), found increased serum TNF-α and IL-10 levels (31), again indicating a systemic inflammatory response.

Few studies have addressed the levels of apolipoproteins in protein-energy malnutrition. An early study from Feillet and colleagues reported low plasma total cholesterol, HDL, and LDL-cholesterol, but normal ApoA-I concentration in 39 children, aged between 9 and 44 months, with marasmus. The ApoAI/ApoA-Il ratio did not differ from individuals in the control group, however significantly increased triglyceride-rich particles, lipoprotein C-III:B, and lipoprotein E:B were found (32).

In our study, greater hepatic levels of ApoA-I mRNA were seen in RBD-challenged mice compared to controls, suggesting an increased mobilization of cholesterol from the tissues to the liver. As ApoA-I biosynthesis in the liver may be associated with a response to oxidative stress (33), increased ApoA-I transcriptional levels in undernutrition might be a compensatory response to protect against inflammation derived-free radicals. TNF-α has been shown to downregulate ApoA-I expression in hepatic cell lines, which may be reverted by PPAR-γ inhibitors (34). Our data showed increased liver ApoA-I mRNA levels regardless of the high TNF-α transcriptional activity. The reason for such observation is unknown but may indicate the need for more ApoA-I activity in the liver to overcome the inflammatory insult. Noteworthy is that ApoA-I is able to induce the secretion of IL-10 by monocytes (35) (and perhaps liver macrophages), which may further contribute to reduce excessive inflammation.

LCAT is responsible for the esterification of free cholesterol within HDL particles and its activity is strongly regulated by ApoA-I. In our malnutrition protocol with RBD, no difference was seen in LCAT transcription in the liver, however, we could not rule out a later effect with even worse chronic malnutrition. It has been shown that long-term feeding with hypoproteic diets (with 2% of casein for 28 days) is able to reduce LCAT activity (but not HDL plasma levels nor ApoA-I levels) (36). The discrepancy from our findings may be due to different levels of protein malnutrition in our diet.

Interestingly, the liver from undernourished mice showed hypertrophied and polypoid hepatocytes. Both features have been found with decreased liver function and parasitic burden seen in cryptosporidiosis and might be a consequence of hepatocyte work overload and changes related to epigenetic programming (37). Long-term alterations in the liver cholesterol reverse transport may increase the likelihood of atherosclerotic harmful effects with aging. When malnutrition-related intestinal-to-blood bacterial translocation occurs, production of phospholipid-rich very low-density lipoprotein might be increased by the liver (resulting in hypertriglyceridemia) to neutralize bacterial products with increased accumulation of cholesterol in cells, and if a chronic and repeated insult is present, it may initiate atherosclerotic lesions (38). 

Although no alteration in ALT serum levels was seen nor liver steatosis (suggesting no significant liver dysfunction), altogether our findings indicated an inflammatory liver state (although subclinical) with altered hepatic ApoA-I expression, the latter as a potential compensatory response to protect against increased inflammation. Nonetheless, such malnutrition-related liver long-term changes may have pro-atherosclerotic effects with aging.

One limitation of this study is that the diets may have proteins of different quality, for example, a different amino acid profile and digestibility, which is difficult to be standardized, which may have resulted in different weight gain and biochemical profiles of the experimental animals. However, socially disadvantaged populations in poor regions of Brazil likely face the same problem.

In conclusion, our overall data suggest that the chronic feeding with the RBD caused liver inflammation with increased hepatic ApoA-I expression. These findings may support ApoA-I as a potential therapeutic target in animal models of low-protein malnutrition. More mechanistic studies are warranted to further confirm and extend our results.
